# SOCS3 inhibits the mesenchymal stromal cell secretory factor SDF-1-mediated improvement of islet function in non-obese diabetic mice

**DOI:** 10.1186/s13287-023-03347-y

**Published:** 2023-07-03

**Authors:** Mingxing Sui, Tuo Li, Hanlan Lu, Yanhua Li, Juan Huang, Pei Zhang, Shusen Wang, Li Zeng

**Affiliations:** 1 Department of Organ Transplantation, Shanghai Changhai Hospital, Navy Medical University, 168 Changhai Road, Shanghai, 200433 China; 2 Department of Endocrinology, Changzheng Hospital, Navy Medical University, 415 Fengyang Road, Shanghai, 200003 China; 3 Organ Transplant Center, Tianjin First Central Hospital, Tianjin, China

**Keywords:** CXCR4, Islet transplantation, SDF-1, SOCS3, Type 1 diabetes

## Abstract

**Background:**

Islet transplantation is used therapeutically in a minority of patients with type 1 diabetes (T1D). However, successful outcomes are hampered by early islet β-cell loss caused by immune rejection and autoimmunity. Recent studies have demonstrated that mesenchymal stromal cells can enhance islet function both in vitro and in vivo by secreting ligands that activate islet G-protein coupled receptors (GPCRs). Stromal cell-derived factor 1 (SDF-1) is an MSC-secreted GPCR ligand, whereas the suppressor of cytokine signaling 3 (SOCS3) is a negative regulator of STAT3-activating cytokines. Here, we determined whether improvement in islet function mediated by exogenous SDF-1 is impaired by SOCS3 in experimental models of T1D.

**Methods:**

Isolated islets were cultured for 48 h with SDF-1. Cytokine-induced apoptosis was measured immediately. Islets from *Socs3*^−/−^ mice were pre-cultured with exogenous SDF-1 and transplanted underneath the kidney capsule of C57BL/6 mice with streptozotocin-induced diabetes. Blood glucose levels were monitored for 28 days. AMD3100, an antagonist of the SDF-1 ligand CXCR4, was administered subcutaneously to islet transplanted mice to inhibit CXCR4 before and after transplantation.

**Results:**

SDF-1 protected islet cells from cytokine-induced apoptosis in vitro. SOCS3-knockout (KO) islets pretreated with SDF-1 were effective in reducing blood glucose in non-obese diabetic mice in vivo. We found that SDF-1 elicits localized immunosuppression in transplanted SOCS3-KO islets. Immunomodulation was observed when SOCS-KO islets were preconditioned with SDF-1. Gene expression and flow cytometric analyses revealed significantly decreased immune cell infiltration, inflammatory cytokines, and concomitant increases in FOXP3^+^ regulatory T cells, alternatively activated M2 macrophages, and dendritic cell phenotypes. Administration of AMD3100 impaired the SDF-1-mediated improvement in SOCS3-KO islet function and local immune suppression.

**Conclusion:**

SDF-1 improves the function of islet grafts in autoimmune diabetes through regulation by CXCR4; however, the presence of SOCS3 reverses the protective effect of SDF-1 on islet grafts. These data reveal a molecular pathway that can elicit localized immunosuppression and delay graft destruction in transplanted islets.

**Supplementary Information:**

The online version contains supplementary material available at 10.1186/s13287-023-03347-y.

## Introduction

Type 1 diabetes (T1D) is predominantly the result of autoimmune-related injury to insulin-producing pancreatic islet β-cells of Langerhans [1, 2]. The consequential glycemic variability requires lifelong insulin management and leads to increased rates of morbidity through cardiovascular events and other clinical outcomes [3, 4]. Islet transplantation offers an effective and natural means of managing glucose levels by restoring insulin secretion in situ with fewer complications than whole pancreas transplantation. However, several factors impede its success and lead to excessive islet loss and failure to secrete sufficient insulin [5, 6]. For instance, immediately following transplantation, islets are confronted with a hypoxic and inflammatory host environment that often leads to their destruction and the failure of the grafting process and the number of surviving islets fail to secrete sufficient insulin to normalize hyperglycemia [7, 8]. The cotransplantation of mesenchymal stromal cells (MSCs) improves islet survival following transplantation in animal models [9]. MSCs were found to inhibit the autoimmune destruction of islets through the induction of IL10-secreting T cells and the suppression of diabetogenic T cell infiltration [10]. The secretomes of MSCs are believed to modulate the host environment by suppressing immune responses and enabling the activation of G-protein coupled receptors (GPCRs) in the transplanted islets to improve the secretion of insulin [11–13].

Stromal cell-derived factor 1 (SDF-1, alias CXCL12) is a GPCR ligand that promotes β-cell production and is highly expressed in β-cells during injury and regeneration [14]. SDF-1 is abundant in the MSC secretome where it is associated with the repair of damaged tissue and the modulation of immune responses [15, 16]. Islets that are pre-cultured with MSC-derived SDF-1 are protected from cytokine-induced apoptosis [13]. CX chemokine receptor 4 (CXCR4) is a ligand of SDF-1 that is expressed on the surface of CD4^+^ T cells and is upregulated in injured islets [17]. SDF-1 has been found to protect non-obese diabetic (NOD) mice from autoimmune diabetes through the recruitment of CXCR4^+^ T cells [18]. The balance between various T cells is found to be a contributory factor in the development of diabetes in the NOD mouse model [19].

In this study, we investigated whether the regulation of CXCR4 by exogenous SDF-1 may protect islet grafts from the autoimmune response. Moreover, we examined the relationship between the CXCR4/SDF-1 axis and the suppressor of cytokine signaling 3 (SOCS3). SOCS3 is a negative regulator of STAT3-activating cytokines that is believed to regulate cytokine-induced apoptosis in β-cells [20, 21]. The overexpression of SOCS3 inhibits T-helper cell proliferation, whereas SOCS3 silencing enhances T cell receptor and cytokine-induced proliferation [20]. In this study, we investigated the role of SOCS3 in islet survival. We believe that SOCS3 may reverse the protection offered by SDF-1 in transplanted islets because it is proposed to subdue the JAK/STAT pathway and SDF-1 by binding to CXCR4 [22]. CD4^+^ T cells expressing FOXP3 are thought to promote immune tolerance in β-cells and, therefore, increased levels of FOXP3^+^ CD4^+^ T cells signify a reduction in autoimmunity [23]. Consequently, in this study, we measured immunomodulatory responses in SOCS3-knockout (KO) islets preconditioned with SDF-1 and levels of FOXP3^+^ regulatory T cells. Our data revealed a molecular pathway that can elicit localized immunosuppression and has the potential to delay graft destruction in human islet transplantation.

## Materials and methods

### Experimental animals

Male C57Bl/6 mice aged 8–12 weeks were used as islet donors for all in vitro investigations. SOCS3-KO (*Socs3*^−/−^) and wild-type (WT) male mice on a C57BL/6 J background were obtained from the Shanghai Model Organisms Center, INC, and used as islet donors and recipients, respectively. All animal procedures were conducted and reported according to ARRIVE (Animal Research: Reporting of In Vivo Experiments) guidelines and approved by the Committee of Shanghai Tenth People’s Hospital. Intraperitoneal streptozotocin (STZ, 180 mg/kg; Sigma-Aldrich, St Louis, MO, USA) was used to induce diabetes in male WT mice. Diabetes was confirmed if the blood glucose levels were ≥ 16.7 mM/L for three consecutive days [24]. Renal subcapsular islet transplantations were carried out as described previously [25]. Briefly, a lumbar incision was made to expose the kidneys and 200 islets were transplanted into mice under the kidney capsule. Preconditioned islets were pre-cultured with 10 nmol/L SDF-1 for 2 days before transplantation. To inhibit CXCR4, and subsequently SDF-1, mice were administered with a CXCR4 antagonist, AMD3100 (Sigma-Aldrich) [26], subcutaneously at 10 mg/kg in 200 µl of phosphate-buffered saline (PBS) three times a week 1 week before islet transplantations and every day for 2 weeks thereafter. Mice were euthanized with an intraperitoneal injection of saline-diluted Euthasol (150 mg/kg; Virbac Animal Health) containing pentobarbital sodium (390 mg/mL) and phenytoin sodium (50 mg/mL) as the active ingredients. Briefly, the mice were picked up by hand, scruffed, and inverted; the mouse's head was down at a slight (approximate 20°) angle, and the injection was given in the lower left abdominal quadrant.

### Graft function in vivo

Graft function in vivo was assessed as described previously [27]. Non-fasting blood glucose concentrations and body weight were measured every 3 days. Two consecutive measurements of blood glucose < 16.7 mM/L were considered to be a reversal of hyperglycemia [28]. An intraperitoneal glucose tolerance test (IPGTT) was conducted 28 days after transplantation in mice with a blood glucose concentration < 16.7 mM/L.

### Immunofluorescence

Specimens containing kidney islet allografts were fixed with 4% paraformaldehyde and processed for paraffin embedding. Polyclonal mouse anti-insulin antibody (#8138; Cell Signaling Technology, Danvers, MA, USA), rabbit monoclonal to CD4 (ab183685; Abcam, Cambridge, MA, USA), and rabbit monoclonal to CD8 (ab217344; Abcam) were used as primary antibodies. Goat anti-mouse IgG Alexa Fluor 488 (Invitrogen, Carlsbad, CA, USA) and goat anti-rabbit IgG Alexa Fluor 555 (Invitrogen) were used as secondary antibodies. Slides were examined on a Fluorescence Microscope.

### Flow cytometry analysis

Flow cytometry was performed as described previously [29]. Briefly, excised kidney islets were homogenized to create a cell suspension and resuspended at approximately 2 × 10^7^ cell/mL. Cells were identified in different experimental groups using fluorescent-conjugated antibodies. Cells were fixed and stained using BD Cytofix (BD Biosciences, Franklin Lakes, NJ, USA) according to the manufacturer’s instructions and quantified with an Attune NxT Flow Cytometer (Thermo Fisher Scientific, Waltham, MA, USA). Results were analyzed using FlowJo v.10.

### Quantitative real-time (RT)-PCR analysis

Total RNA was isolated from islet grafts with TRIzol (Life Technologies, Carlsbad, CA, USA). Reverse transcription and quantitative (qRT)-PCR was performed using commercially available reagents (Toyobo, Osaka, Japan). A StepOne Real-Time PCR System (ABI, Foster City, CA, USA) was used to detect IL-2, IFN, IL-4-γ, IL-6, and Socs3. β-Actin served as a control. The following primer sequences were used for qRT-PCR:β-actin: forward 5′-CATCCGTAAAGACCTCTATGCCAAC-3′ and reverse 5′-ATGGAGCCACCGATCCACA-3′; IL-2: forward 5′-GGAGCAGCTGTTGATGGACCTAC-3′ and reverse 5′-AATCCAGAACATGCCGCAGAG-3′; IFN-γ: forward 5′-CGGCACAGTCATTGAAAGCCTA-3′ and reverse 5′-GTTGCTGATGGCCTGATTGTC-3′; IL-6: forward 5′-GGCCCTTGCTTTCTCTTCG-3′ and reverse 5′-ATAATAAAGTTTTGATTATGT-3′; Socs3: forward 5′-TGAGCGTCAAGACCCAGTCG-3′ and reverse 5′-CACAGTCGAAGCGGGGAACT-3′.

### Enzyme-linked immunosorbent assay (ELISA)

ELISA was carried out to determine inflammatory responses by measuring serum cytokine expression levels (IFN-γ, IL-6, and IL-2). Serum was obtained from blood collected from each treatment group and stored at − 80 °C in aliquots. ELISA kits were obtained from Invitrogen and Cusabio Biotech (Wuhan, China), and the procedure was performed following the manufacturer's protocol. Standard curves were drawn, and the concentration of cytokines was calculated relative to the standards.

### Islet isolation and culture

Islets were isolated by type XI collagenase digestion as described previously [27]. A density gradient (Histopaque-1077, Sigma-Aldrich) was used to separate the islets from the suspension. The isolated islets (100 per group) were cultured alone in RPMI-1640 or pre-cultured with 10 nmol/L SDF-1 for 2 days before transplantation.

### Islet apoptosis in vitro

Islets were pre-cultured alone or with 5 or 10 nmol/L SDF-1 for 48 h and then cultured with mixed cytokines (50 U/mL IL-1β, 1000 U/mL IFN-γ, and 1000 U/mL TNF-α) for the final 20 h of incubation. Islets cultured without cytokines served as controls. TdT-mediated dUTP nick-end labeling (TUNEL) analysis was used to evaluate β-cell apoptosis as described previously [30]. Islets were assayed on poly-l-lysine slides using an ApopTag in situ detection kit (Millipore, Burlington, MA, USA). Images were obtained using a confocal laser-scanning microscope (FluoView FV1000-D, Olympus, Tokyo, Japan).

### Statistical analysis

All experiments were performed independently at least three times. Data are expressed as the mean ± standard error of the mean (SEM). Comparisons were performed using the unpaired Student’s *t*-test. Data were analyzed using SPSS 21 (SPSS, Chicago, IL, USA). *p* < 0.05 was considered statistically significant.

## Results

### SDF-1 prevents islet apoptosis in response to cytokines in vitro

**Fig. 1:**
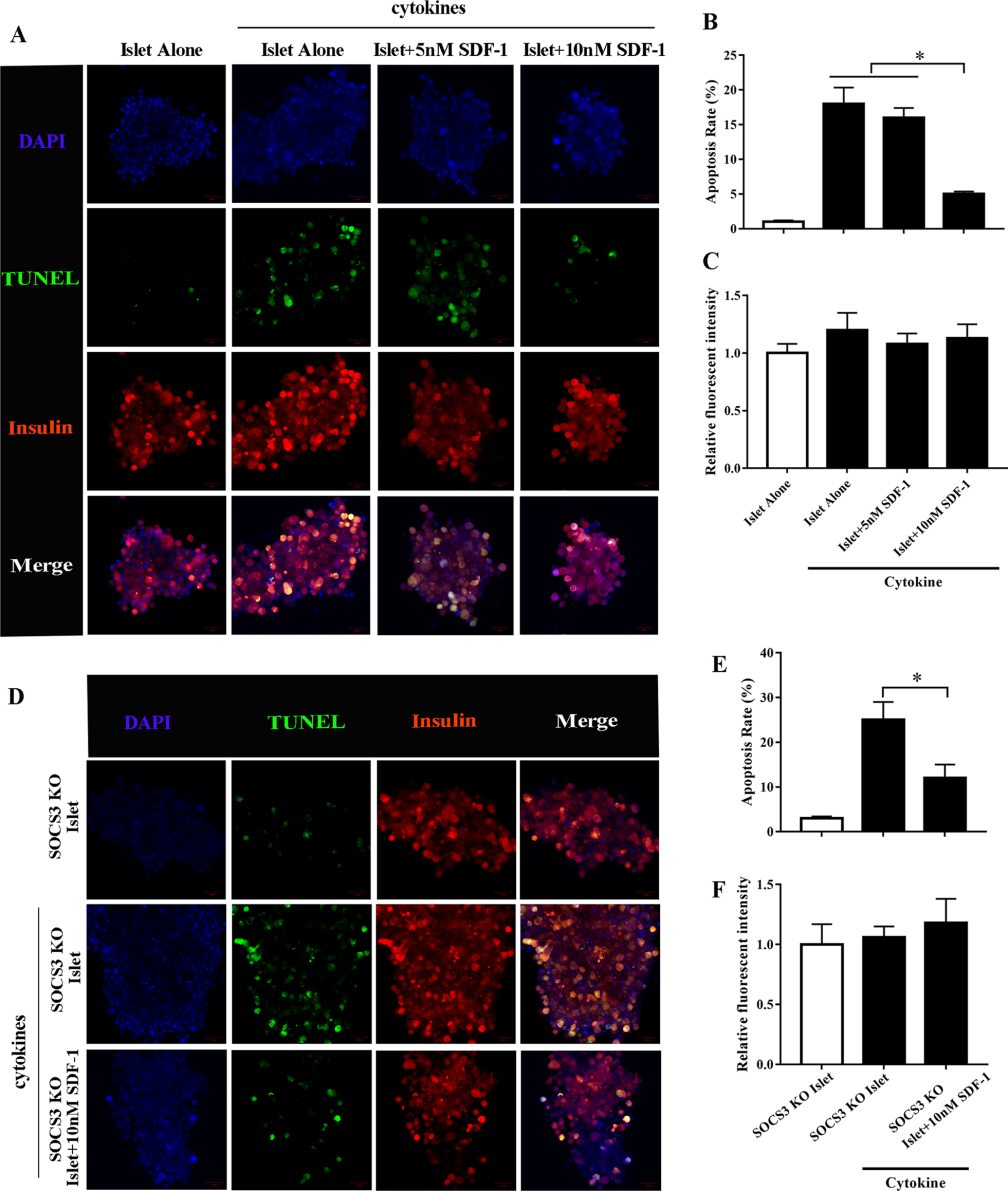
Effects of SDF-1 on islet function in vitro. Islets were pre-cultured with exogenous stromal cell-derived factor-1 (SDF-1). **A**, **B** Dose-dependent protection from cytokine-induced apoptosis following 48 h pre-culture with SDF-1 and the subsequent presence of SDF-1 during the final 20 h cytokine incubation in WT islets. **C** Quantification of insulin staining by MetaMorph analysis in wild-type (Wt) islets. **D**, **E** Pre-culturing SOCS3 knockout (KO) islets with 10 nM SDF-1 protects from cytokine-induced apoptosis. **F** Quantification of insulin staining by MetaMorph analysis in SOCS3 KO islets. Magnification: × 200. *p < 0.05

To establish whether SDF-1 could influence the loss of islets in response to cytokines, we used immunofluorescence and a TUNEL assay to determine the rate of apoptosis. When islets were cultured with 10 nM SDF-1 for 48 h and then cultured with mixed cytokines for the final 20 h of incubation significant protection against apoptosis was observed (Fig. 1A and 1B). The apoptosis rate after the addition of cytokines is the greatest in islets that have not been cultured with SDF-1. A lower concentration of SDF-1 (5 nM) did not have the same impact on the apoptotic rate as the higher concentration (10 nM). There was no significant difference in insulin levels between the groups (Fig. 1C). A similar trend was observed in SOCS3 KO islets (Fig. 1D–F). These results indicate that SDF-1 at a concentration of 10 nM can prevent the apoptosis of islets in response to cytokines.

### SOCS3 is upregulated in islet cells pre-cultured with SDF-1

**Fig. 2:**
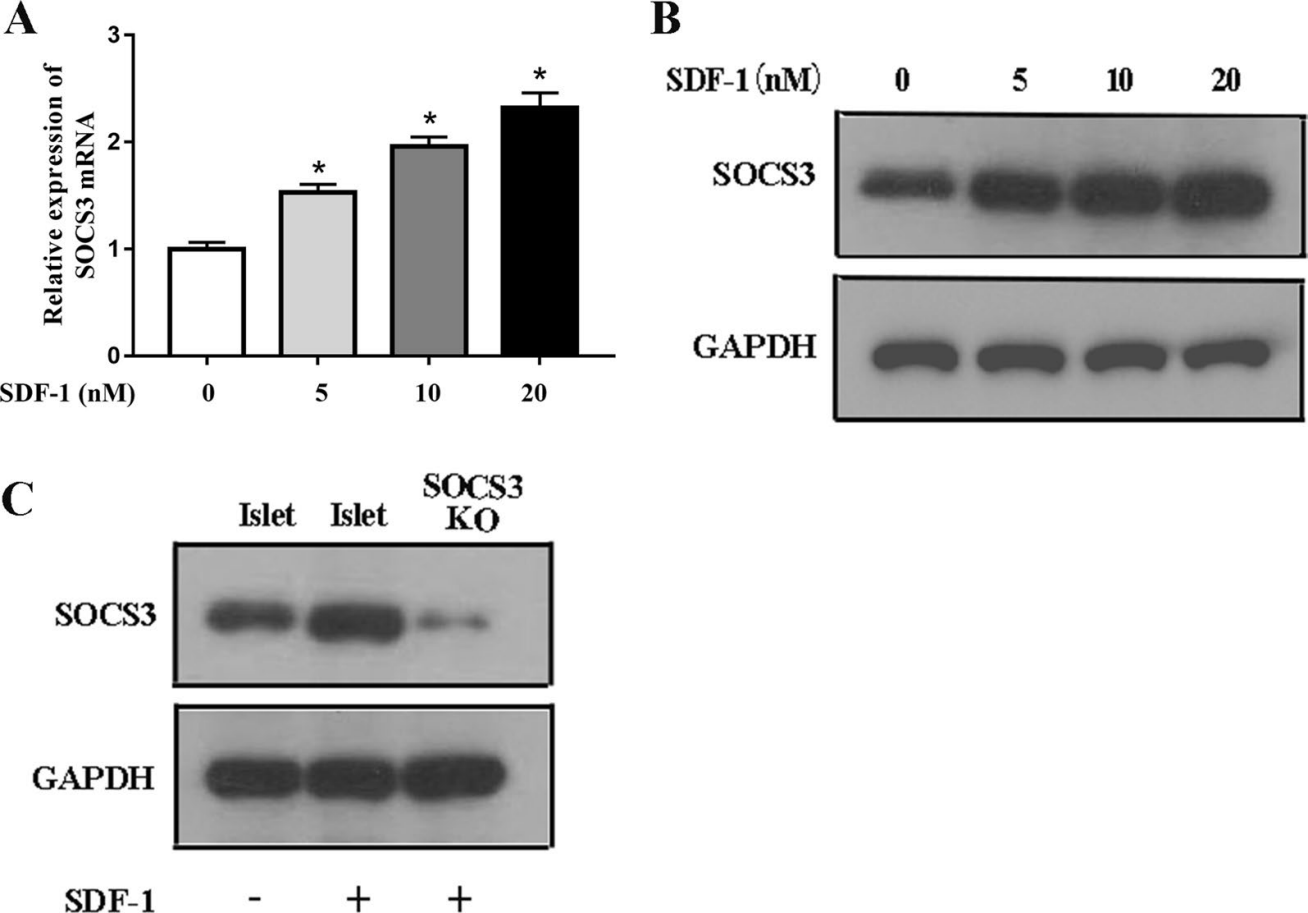
SOCS3 upregulation in islet cells following pre-culture with SDF-1. SDF-1 treatment induces SOCS3 expression in vitro. **A**, **B** SOCS3 mRNA and protein levels were analyzed in islet cells following 48 h pre-culture with 0, 5, 10, 20 nM SDF-1. **C** Western blot analysis of SOCS3 levels in SOCS3 KO and Wt islets following 48 h pre-culture with or without 10 nM SDF-1. *p < 0.05 versus 0 nM SDF-1

To determine the effect of SDF-1 on the expression of SOCS3, we measured the levels of SOCS3 mRNA in islets that were pre-cultured with various concentrations of SDF-1 (0–20 nM). The addition of SDF-1 significantly induces the expression of SOCS3 and the changes in expression are concentration dependent, with the highest expression attained at 20 nM SDF-1 (Fig. 2A). Western blot analysis confirmed that SOCS3 protein levels increased with SDF-1 concentration and that no expression was observed when SOCS3 was knocked down (Fig. 2B and C). These results confirm that the expression levels of SOCS3 are upregulated in islets pre-cultured with SDF-1 (Additional file 1).

### SOCS3 obstructs the improved islet function following pre-culture with SDF-1 in NOD mice

**Fig. 3:**
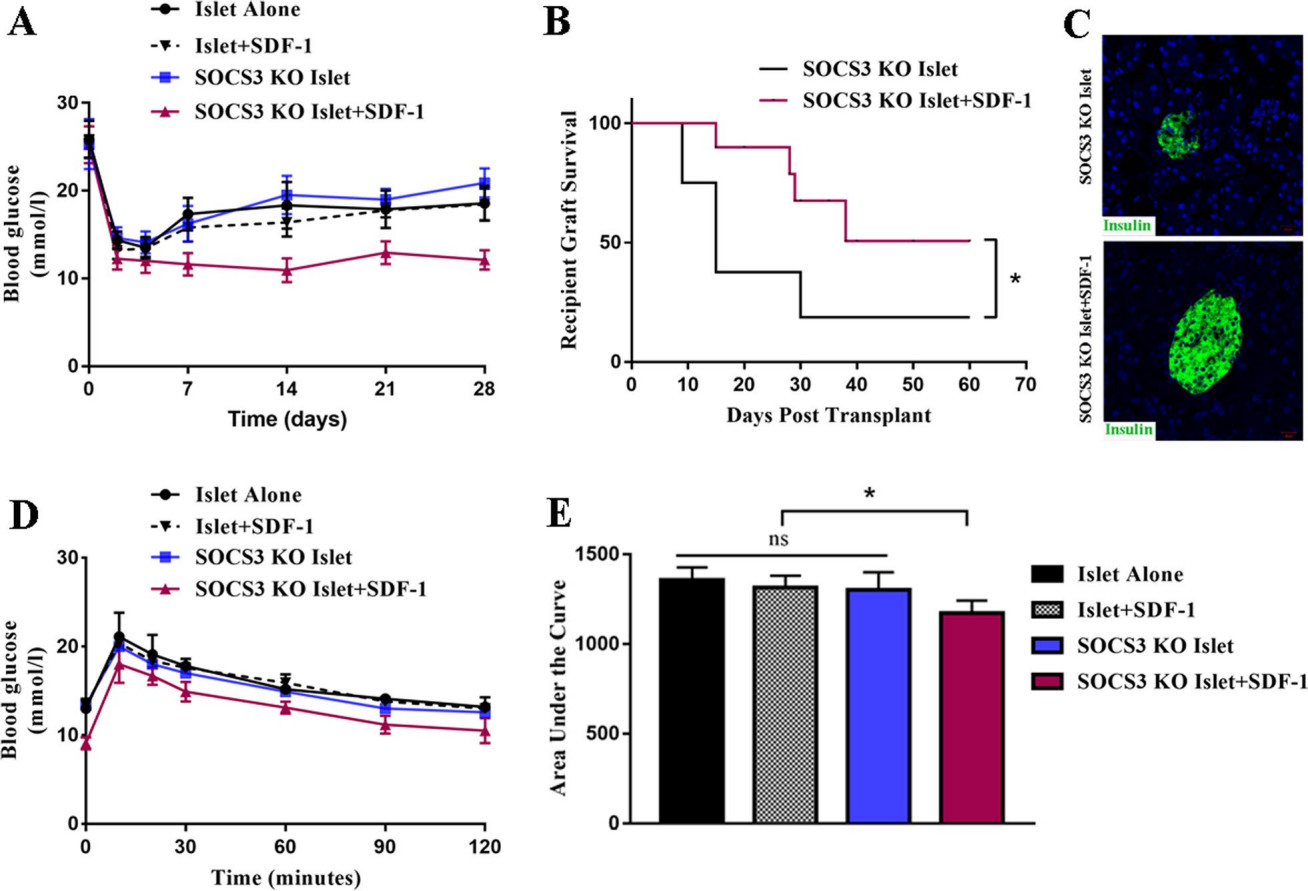
SOCS3 blocks the improvement in islet function following pre-culture with SDF-1 in NOD mice. In vivo graft function of SDF-1 pre-cultured SOCS3-KO islets. **A** Blood glucose concentrations of mice transplanted with 200 islets or SOCS3-KO islets pre-cultured alone or with 10 nM/L SDF-1 for 2 days. SOCS3 KO islets + SDF-1 group showed significantly lower glucose levels compared to those in the other groups (repeated measurement of ANOVA with Bonferroni post hoc test, *n* = 6). **B** Kaplan–Meier Log-rank test of STZ-treated NOD recipients maintaining islet graft function based on blood glucose readings (< 16.7 mM/L) after transplantation with 200 SOCS3-KO islets under the kidney capsule with or without SDF-1 (*n* = 9 per group). **C** Double immunofluorescence staining for DAPI (blue) and insulin (green) at 7 days post-transplantation in SOCS3 KO islets and SOCS3 KO islets + SDF-1 groups. Magnification: × 200. **D** Intraperitoneal glucose tolerance tests (IPGTTs) in all cured mice at 1 month after transplantation as above (repeated-measurement ANOVA with Bonferroni post hoc test, *n* = 3–5). **E** Two-way ANOVA with multiple comparisons for the area under the curve of the IPGTT. ns = not significant; *p < 0.05

After establishing that SDF-1 protects islets in vitro, we determined whether similar results could be obtained in vivo. NOD mice were transplanted with WT islets or islets from *Socs3*^−/−^ mice that were pre-cultured with or without SDF-1 (10 nM). Although SDF-1 preconditioning reduces cytokine-induced β-cell apoptosis in vitro, pretreatment with SDF-1 does not significantly protect transplanted islets in vivo. After 28 days, blood glucose levels were similar in mice transplanted with islets cultured with or without SDF-1 (Fig. 3A). However, blood glucose levels were significantly lower in mice transplanted with SOCS3-KO islets that were cultured with SDF-1, but glucose levels did not improve in mice transplanted with SOCS3 KO islets that had not been pretreated with SDF-1 (Fig. 3A). Moreover, graft survival improved in mice transplanted with SOCS3-KO islets that were cultured with SDF-1 compared with SOCS3 KO islets that were not pre-cultured with SDF-1 (Fig. 3B and C). These results were confirmed by intraperitoneal glucose tolerance tests (IPGTTs) 1 month after transplantation (Fig. 3D and E). The combined results suggest that the ability of SDF-1 to protect islets is comprised by the presence of SOCS3, indicating that SOCS3 hinders the protective effect of SDF-1 in transplanted islets.

### AMD3100 inhibits improved SDF-1-mediated islet function in transplanted SOCS3-KO islets in vivo

**Fig. 4:**
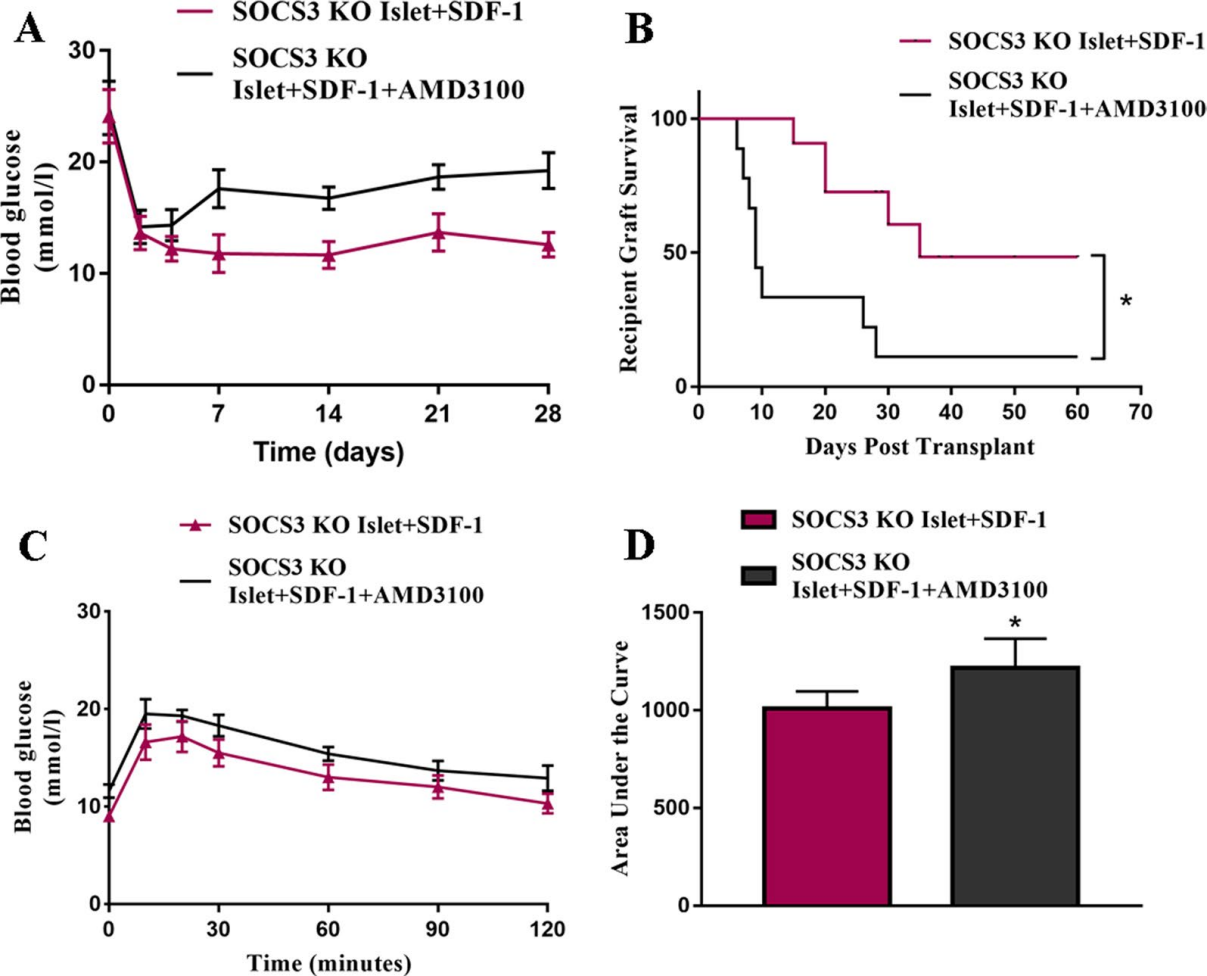
In vivo AMD3100 administration reverses improved graft function of SDF-1 pre-cultured SOCS3-KO islets. **A** Blood glucose concentrations of mice transplanted with SOCS3-KO islet pre-cultured with 10 nM/L SDF-1 for 2 days, and with or without AMD3100 administration (repeated-measurement ANOVA with Bonferroni post hoc test, *n* = 6). **B** Kaplan–Meier Log-rank test of STZ-treated NOD recipients maintaining islet graft function based on blood glucose readings (< 16.7 mM/l) after transplantation with SDF-1 pre-cultured SOCS3-KO islet under the kidney capsule with or without AMD3100 administration (*n* = 10 per group). **C** Intraperitoneal glucose tolerance tests (IPGTTs) in all cured mice at 1 month after transplantation as above (repeated-measurement ANOVA with Bonferroni post hoc test, *n* = 3–5). **D** Two-way ANOVA with multiple comparisons for the area under the curve of the IPGTT. *p < 0.05

To confirm whether the SDF-1-mediated improvement of SOCS3-KO islet function is dependent on CXCR4, we assessed the influence of AMD3100 on graft function. Blood glucose levels were significantly lower in mice that received SOCS3-KO islets pre-cultured with SDF-1 than in mice that received the same graft but were administered AMD3100 (Fig. 4A). Graft survival was also decreased in mice that received AMD3100 (Fig. 4B). IPGTTs confirmed increased glucose levels and graft failure in the mice treated with AMD3100 (Fig. 4C and D). These results verify that the improvement in SOCS3-KO islet grafts in NOD mice was mediated by SDF-1–CXCR4 binding because AMD3100 could obstruct islet function through the inhibition of CXCR4.

### SDF-1 can elicit localized immunosuppression in transplanted SOCS3-KO islets

**Fig. 5:**
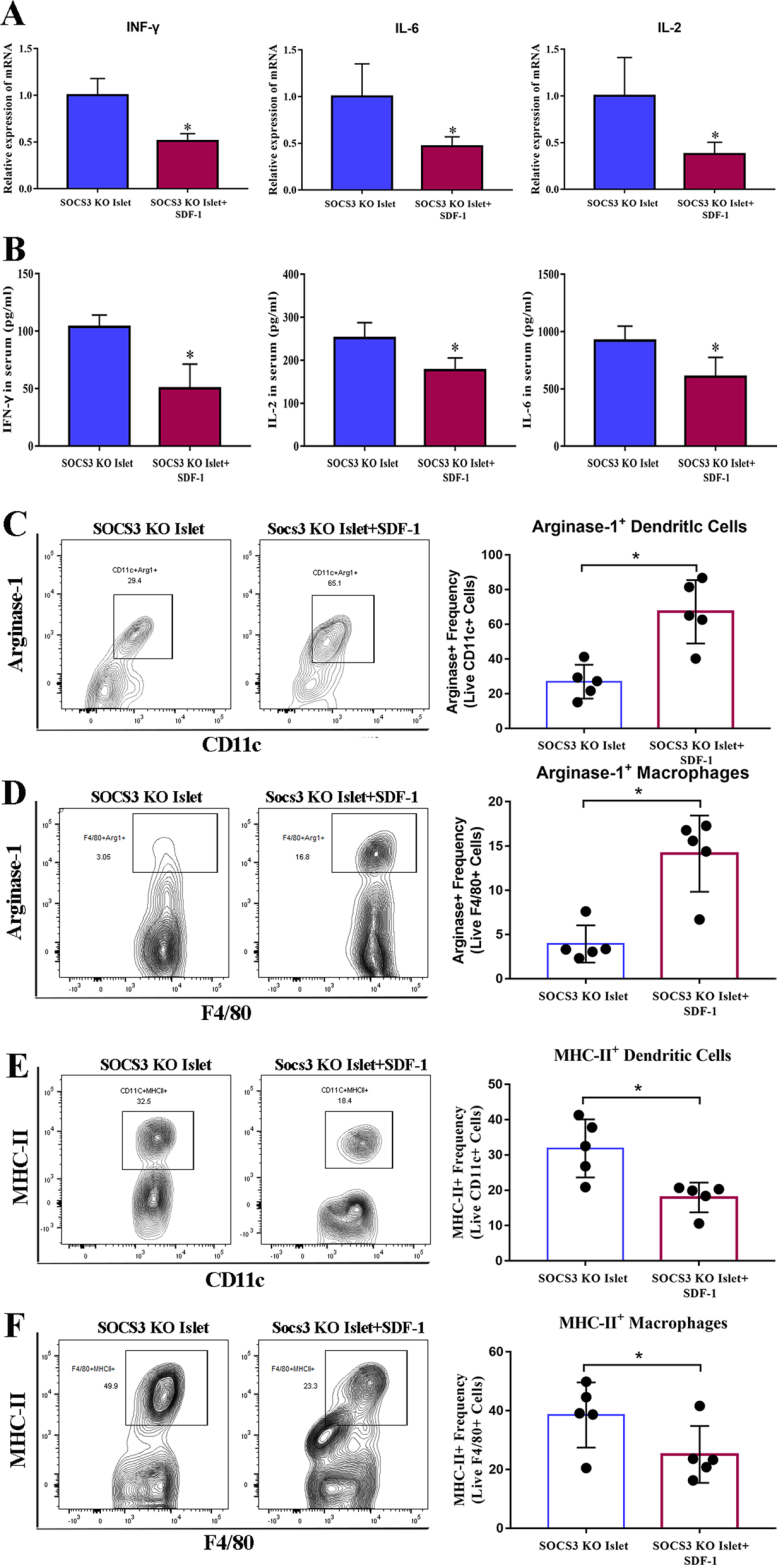
SDF-1 preconditioning suppressed inflammatory responses in the grafted SOCS3-KO islets. SOCS3-KO islet allografts with or without SDF-1 were excised from STZ-treated NOD mice and gene expression and flow cytometry were performed (day 7). Graft IFN-γ, IL-2, and IL-6 mRNA levels were quantified by qRT-PCR (**A**), and protein concentrations in recipient mouse sera were determined by ELISA (**B**). Flow cytometry analysis of the frequency of arginase-1^+^ CD11c^+^ DCs (**C**), arginase-1^+^ F4/80^+^ macrophages (**D**), MHC-II^+^ CD11c^+^ DCs (**E**), and MHC-II^+^ F4/80^+^ macrophages (**F**) (*n* = 5). Data represent three independent experiments. *p < 0.05

To investigate whether SDF-1 influenced the inflammatory response we measured levels of IFN-γ, IL-2, and IL-6 mRNA in islet allografts excised from STZ-treated NOD mice. The SOCS3-KO islets that had been preconditioned with SDF-1 had significantly lower levels of IFN-γ, IL-2, and IL-6 mRNA expression than those that had not been preconditioned (Fig. 5A), suggesting that SDF-1 could suppress inflammatory responses in SOCS3-KO islets because it is not inhibited by the actions of SOCS3. These results were confirmed by ELISA (Fig. 5B). Similarly, flow cytometry revealed that arginase-1^+^ CD11c^+^ dendritic cells (DCs) and arginase-1^+^ macrophages were found at higher ratios in SDF-1 preconditioned SOCS3-KO islets, whereas the ratio of MHC-II^+^ CD11c^+^ DCs and MHC-II^+^ macrophages were lower than in SOCS3-KO islets that had not been preconditioned (Fig. 5C–F). This indicates that SDF-1 promotes an alternatively activated macrophage phenotype, suppress chemokine synthesis, and reduce MHC-II expression of innate immune cells in SOCS3-KO islets.

### SDF-1 abrogates T cell infiltration in transplanted SOCS3-KO islets

**Fig. 6:**
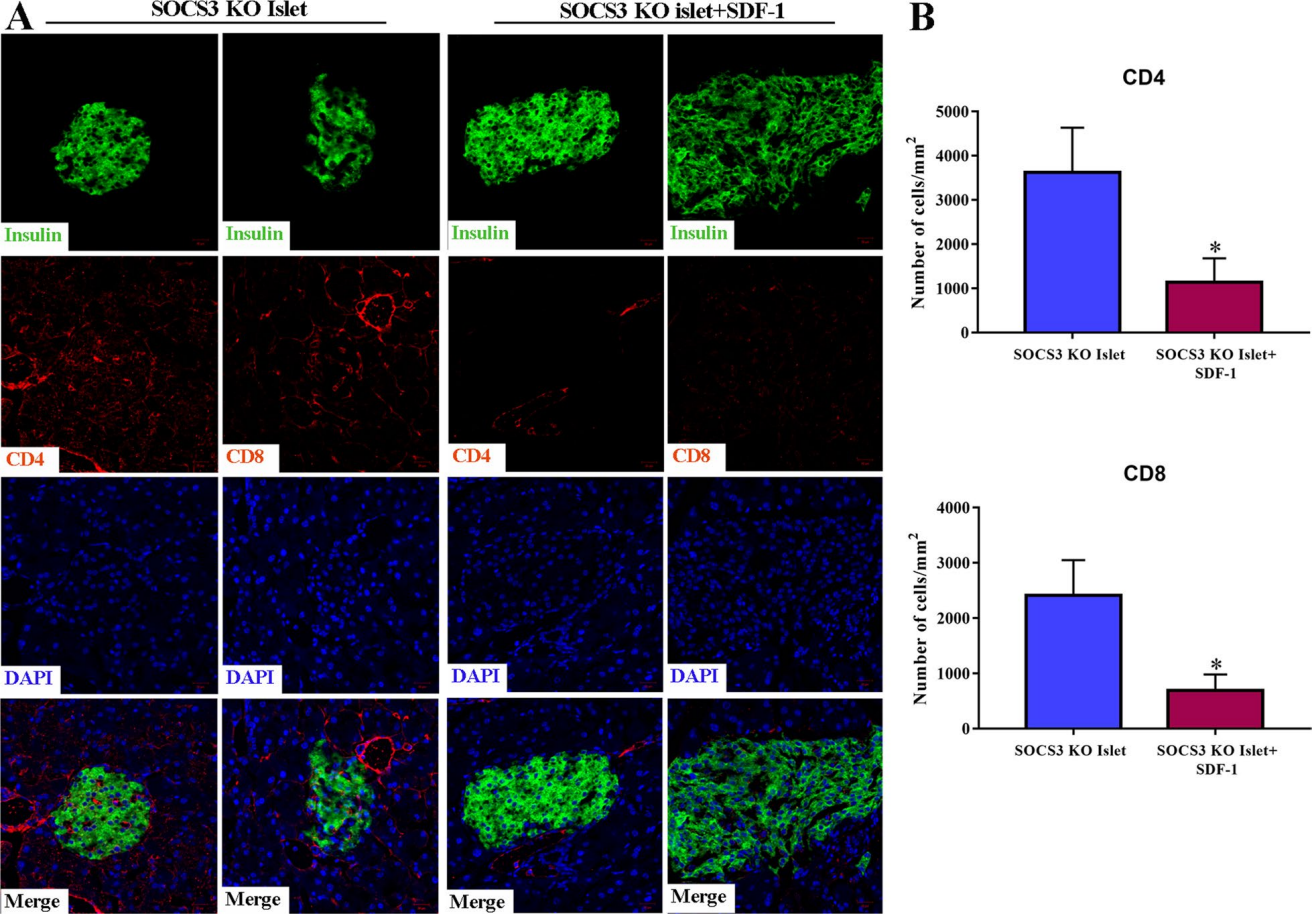
SDF-1 abrogate T cell infiltration in transplanted SOCS3-KO islets. Kidney islet allografts were excised 40 days post-transplant, sectioned, and then stained with immunofluorescence to detect CD4 or CD8 T cells (red), insulin (green), or DAPI in SOCS3-KO islets with or without SDF-1 (**A**). Magnification: × 200. Three fields of vision were assessed for five mice from each group, and the numbers of infiltrating cells of the indicated subtypes were determined (**B**). *p < 0.05

To obtain further evidence, kidney islet allografts were excised 40 days after transplantation and CD4^+^ or CD8^+^ T cells and levels of insulin were stained with immunofluorescence. Infiltration by CD4^+^ or CD8^+^ T cells was significantly reduced in SOCS3-KO islets that were preconditioned with SDF-1 and secretion of insulin appeared to increase (Fig. 6A and B). These results indicate that T cell infiltration is abrogated and islet function is substantially improved by preconditioning SOCS3-KO islets with SDF-1.

### SDF-1 achieves an immunosuppressive effect by pairing with its receptor CXCR4 in transplanted SOCS3-KO islets

**Fig. 7:**
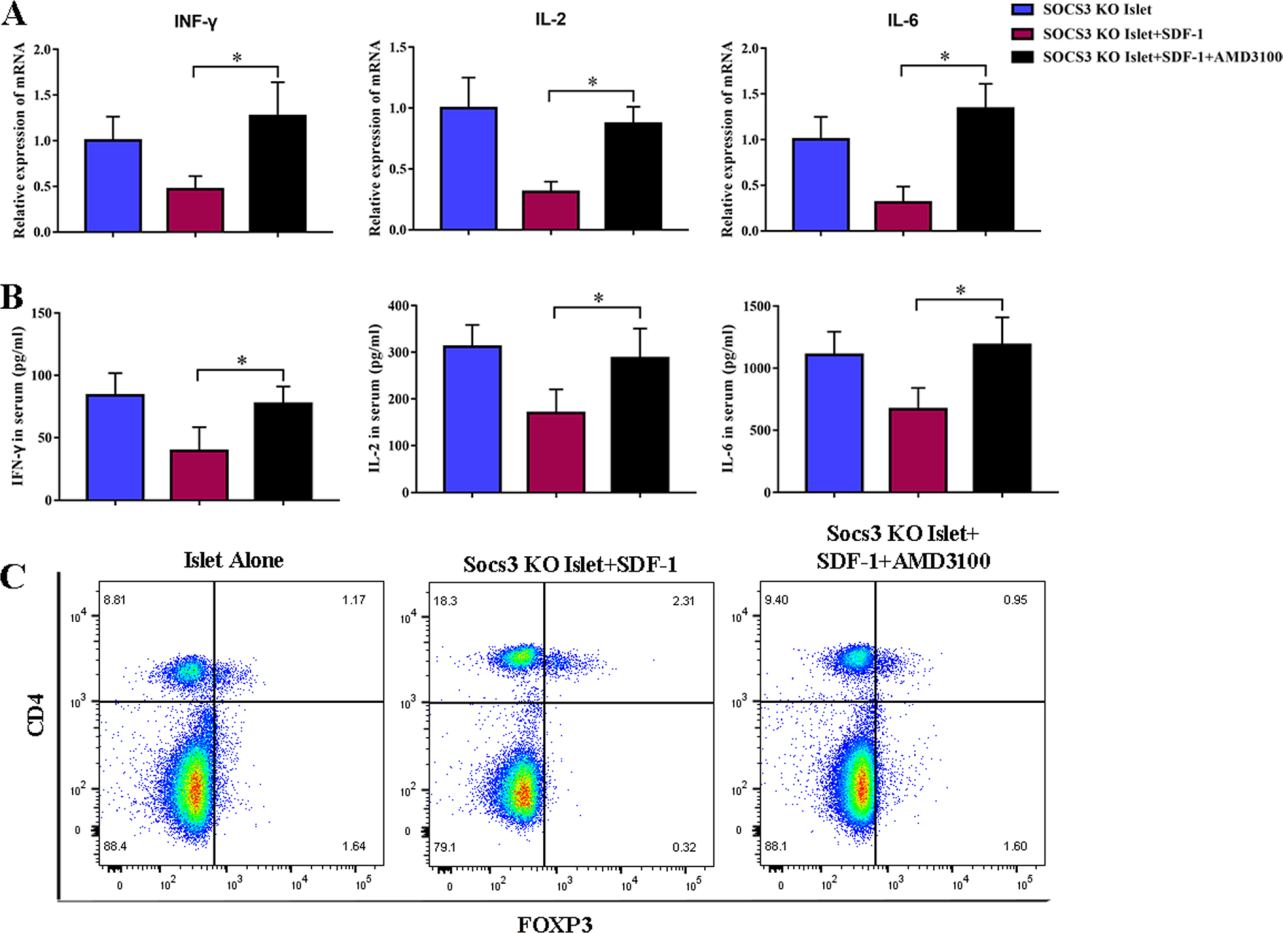
In vivo administration of AMD3100 suppresses SDF-1-mediated immunosuppression in transplanted SOCS3-KO islets. **A** Graft mRNA was analyzed by qRT-PCR for inflammatory cytokines. **B** Protein concentrations in recipient mouse sera were determined by ELISA. **C** Flow cytometry analysis of the frequency of CD4^+^ FOXP3^+^ T cells. *p < 0.05

Finally, we determined whether the in vivo administration of AMD3100 suppresses SDF-1-mediated immunosuppression in transplanted SOCS3-KO islets. AMD3100 was found to suppress SDF-1-mediated immunosuppression of IFN-γ, IL-2, and IL-6 mRNA by binding to CXCR4 in transplanted SOCS3-KO islets (Fig. 7A). Similar results were obtained by ELISA (Fig. 7B). The frequency of FOXP3^+^ regulatory T cells that was increased with SDF-1 preconditioning was decreased with the administration of AMD3100 (Fig. 7C). These results signify that SDF-1 increases immunosuppression and the level of M2 T cells through interactions with its receptor CXCR4.

## Discussion

In contrast to the peripheral cell resistance of endogenous insulin in type 2 diabetes, T1D is mainly the consequence of autoimmune disease, leading to the destruction of the β-cells that produce insulin [31]. The transplantation of islets is viewed as a potential solution but ensuring sufficient islet survival and function in an autoimmune environment is a difficult challenge [5, 32, 33]. The purpose of this study was to investigate ways of prolonging the survival of transplanted islets. To this end, we preconditioned islets with SDF-1, a GCPR ligand that is highly expressed in β-cells during regeneration [14]. In addition, we investigated the combination of SOCS3 deletion and SDF-1 preconditioning on β-cell autoimmunity and allograft rejection. The loss of SOCS3 may subdue the immune responses that cause the dysfunction of β-cells [34]. We found that SDF-1 prevented cytokine-mediated apoptosis of islets in vitro and that increasing concentrations of SDF-1 increased the expression of SOCS3 dose dependently. At first, there was no significant difference in the survival of islets preconditioned with SDF-1 or without preconditioning and transplanted into NOD mice. However, when SDF-1-preconditioned SOCS3 KO islets were transplanted into NOD mice we observed a significantly increased level of islet survival. The protective effect of SDF-1 on the graft may be offset by immune response induced by SOCS3. However, in vitro SDF-1 was effective in reducing cytokine-induced apoptosis due to the lack of influence of the immune microenvironment.

SDF-1 is secreted by MSCs and has been reported to enhance the survival of islets during transplantations [35–38]. For instance, the preconditioning of bone marrow-derived MSCs with SDF-1 has been found to enhance the generation of pancreatic β-cells in an STZ-induced mouse model of diabetes [37]. Research by Rackham et al. shows that pre-culturing islets for 48 h with SDF-1 protected them from inflammatory cytokines in a dose-dependent manner, with a statistically significant effect observed at 10 nM/L SDF-1. The protective effect at this concentration of SDF-1 was reproducible between different islet preparations and increasing the concentration to 20 nmol/L conferred no further protection [13]. Although our study found that SDF-1 (0, 5, 10, and 20 nM) pretreatment induced a linear increase in SOCS3 levels, 10 nM was the most effective protective concentration of SDF-1. In this study, we confirmed that preconditioning with SDF-1 enhanced the survival of SOCS3-KO islets in vivo and prevented cytokine-mediated apoptosis in vitro.

The autoimmunity found in T1D is mediated by CD4^+^ T cells, which are involved in M1 macrophage activation and the recruitment of CD8^+^ cytotoxic T cells [39–41]. The polarization of M1 macrophages by CD4^+^ T cells is associated with increased levels of IFN-γ, TNFα, and IL-1β, which are involved in β-cell destruction, whereas increased expression of TGFβ and IL-10 are associated with M2 macrophages [42]. The NOD mouse model shares many factors with T1D in humans, including autoactivation of CD4^+^ and CD8^+^ T cells [43]. Therefore, in this study we have used islets derived from *Socs3*^−/−^ mice to investigate the influence of SDF-1 preconditioning on islet function and survival in NOD mice. Increased levels of CD4^+^ CXCR4^+^ T cells and higher levels of SDF-1 are believed to protect islets from cytokine-mediated apoptosis [18]. We verified this with our results. CXCR4/SDF-1 increased graft survival by increasing the number of CD4^+^ FOXP3^+^ T cells, indicating a higher level of immune tolerance, whereas the administration of AMD3100 inhibits the SDF-1 interaction with CXCR4, thereby reducing immune tolerance and increasing the apoptosis of islets.

In T1D, the proinflammatory cytokines IL-1 and IFNγ are thought to play dominant roles in the destruction of β-cells. SOCS3 was found to inhibit the production of IL-1 and IFNγ in vitro, implying that it may influence autoimmunity in T1D [21]. However, overexpression of SOCS3 could increase islet graft survival in BALB/c mice with alloxan-induced diabetes but not in NOD mice [21]. Kinjyo et al. found that the loss of SOCS3 led to reduced levels of cytokines in T cells [44]. They suggested that through the modulation of STAT3, SOCS3 could regulate the production of TGF-β1 and IL-10 in CD4^+^ T cells [44]. Therefore, we speculated that the suppression of SOCS3 may offer some protection against graft rejection in T1D by inhibiting the immune response. We found that SOCS3 is upregulated in cells preconditioned with SDF-1 and this upregulation is concentration dependent. However, the presence of SOCS3 was found to block the improvement in islet survival achieved by adding SDF-1. In our study, graft survival improved in mice transplanted with SOCS3-KO islets that were cultured with SDF-1 compared with those that were not cultured with SDF-1. Moreover, blood glucose levels were significantly lower in mice transplanted with SOCS3-KO islets that were cultured with SDF-1, but glucose levels did not improve in mice transplanted with SOCS3-KO islets that had not been pretreated with SDF1. Our results imply that the presence of SOCS3 antagonizes the antiinflammatory protective effects on islets that are elicited by SDF-1. This could be caused by the proposed interactions between CXCR4, the SDF-1 receptor, and SOCS3. Soriano et al. found that SOCS3 binds to CXCR4 to subdue the JAK/STAT pathway and SDF-1 without affecting the expression of cell surface chemokine receptor pathways [22]. They propose that SOCS3 regulates the coordination between the cytokine–chemokine network and selectively blocks certain chemokines without interfering with others. Further investigations involving other chemokines are needed to establish the extent of SOCS3 modulation in T1D.

To validate that the protective effects of SDF-1 preconditioning are dependent on CXCR4 we replicated the experiments with AMD3100, an inhibitor of CXCR4. We found that in NOD mice that were transplanted with SDF-1 conditioned SOCS3-KO islets, blood glucose levels were lower than in those that were administered AMD3100. Moreover, the increase in the frequency of CD4^+^ FOXP3^+^ T cells with SDF-1 preconditioning was reduced with the administration of AMD3100 in SOCS3-KO islets. Disruption of SDF-1–CXCR4 binding by the CXCR4 antagonist AMD3100 accelerates the development of diabetes in adoptive transfer models [18] and induces beta-cell apoptosis [43]. In agreement with our results, Aboumrad et al. found that inhibiting CXCR4 with AMD3100 reduced IL-4 and IL-10 production and prevented the CXCR4/SDF-1 pathway from accelerating autoimmune diabetes in NOD mice [18].

## Conclusions

The preconditioning of islets with MSC-derived SDF-1 improves the function of islet grafts by regulating CXCR4 against autoimmune diabetes. The presence of SOCS3 reverses the protective effect of SDF-1 on islet grafts. Our data indicate that modulation of the SDF-1/CXCR4/SOCS3 axis can elicit localized immunosuppression to prevent graft destruction in islet transplantations.

## Supplementary Information

**Additional file 1.** Uncropped full-length gels and blot results of SOCS3.

## Data Availability

The datasets used and/or analyzed during the current study are available from the corresponding author on reasonable request.

## Abbreviations

T1D: Type 1 diabetes
MSCs: Mesenchymal stromal cells
GPCRs: G-protein coupled receptors
SDF-1: Stromal cell-derived factor 1
SOCS3: Suppressor of cytokine signaling 3
CXCR4: CX chemokine receptor 4
NOD: Non-obese diabetic
IL: Interleukin
IFN: Interferon
TUNEL: TdT-mediated dUTP nick-end labeling
qRT-PCR: Quantitative real-time polymerase chain reaction
IPGTT: Intraperitoneal glucose tolerance test
SOCS3 KO: SOCS3 knockout
Wt: Wild type
STZ: Streptozotocin
DCs: Dendritic cells
