# Supplementary material for: SOCS3 inhibits the mesenchymal stromal cell secretory factor SDF-1-mediated improvement of islet function in non-obese diabetic mice
Source: Stem Cell Res Ther. 2023 Jul 3;14:172. doi: 10.1186/s13287-023-03347-y (PMC10318645; doi:10.1186/s13287-023-03347-y)

fig2B-SOCS3-1

fig2B-SOCS3-2

fig2B-SOCS3-3


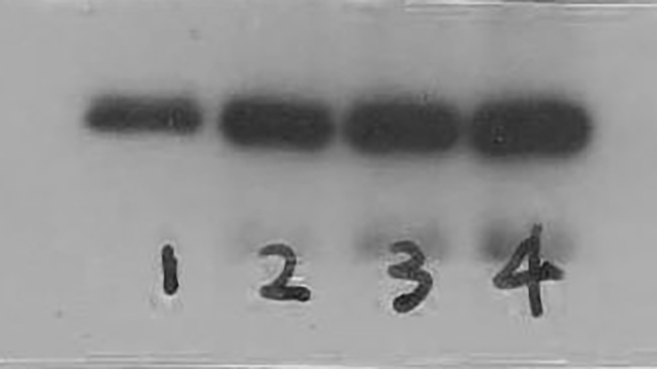

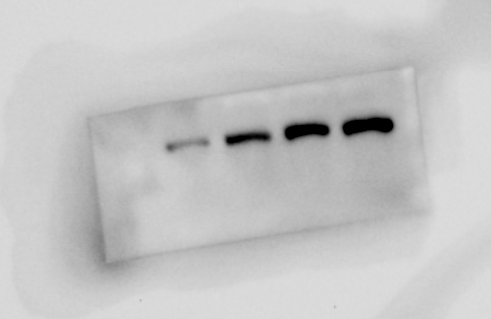

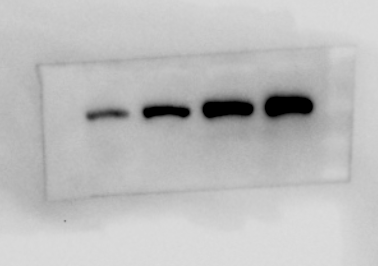


fig2B-GAPDH-1

fig2B-GAPDH-2

fig2B-GAPDH-3


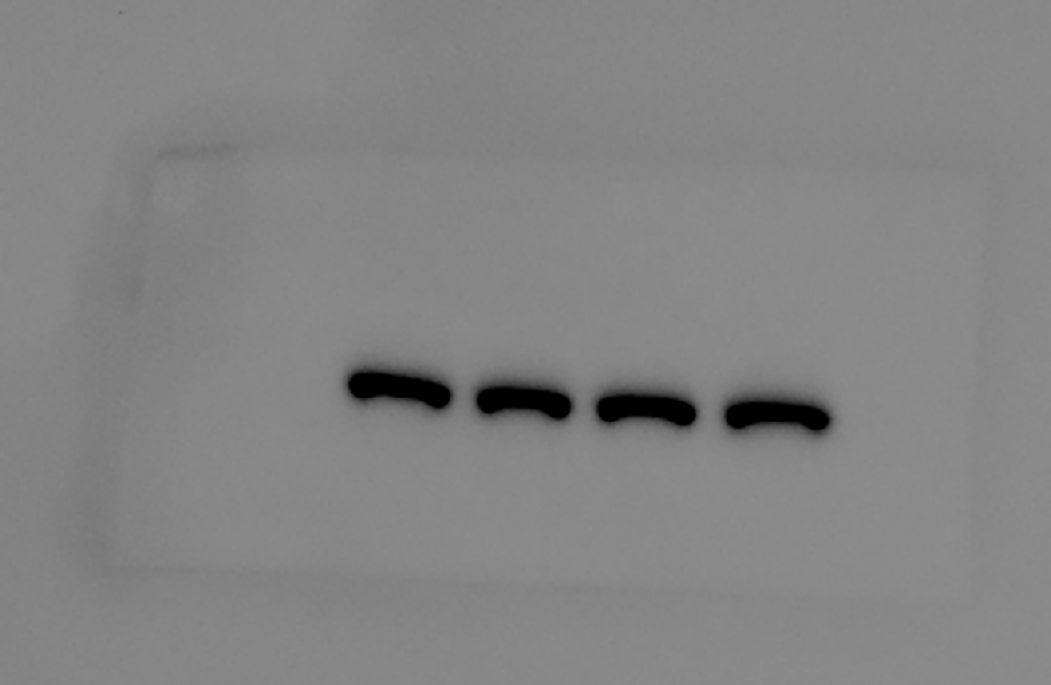

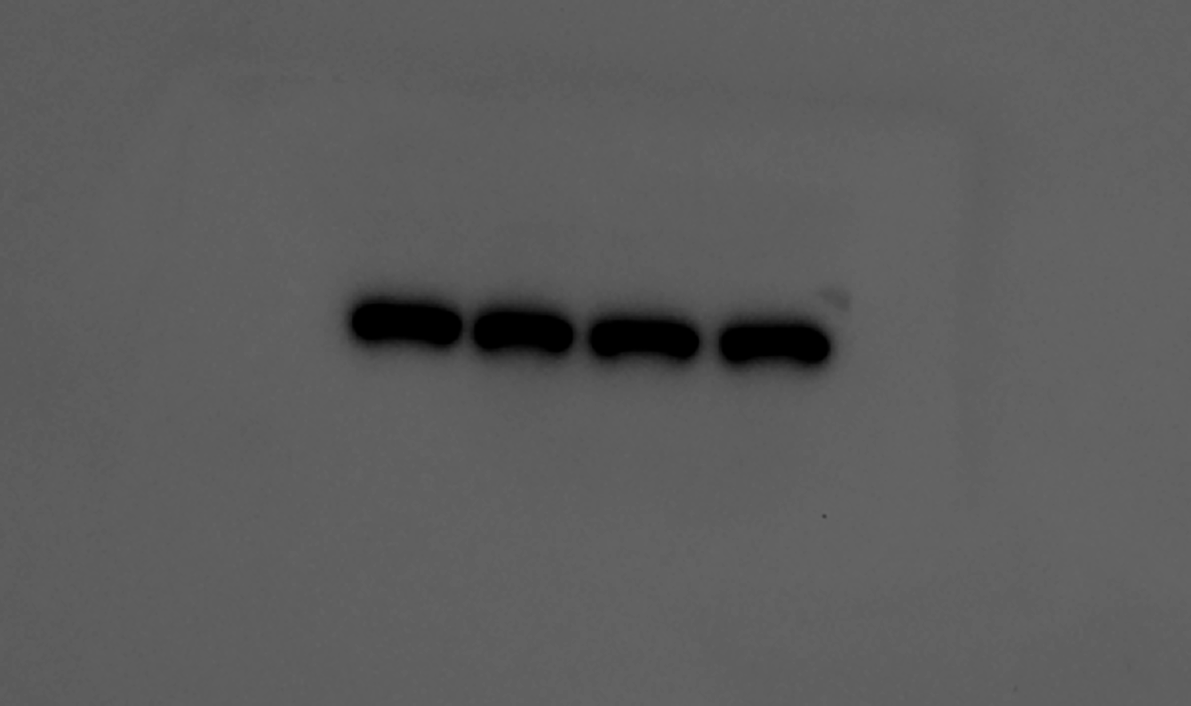

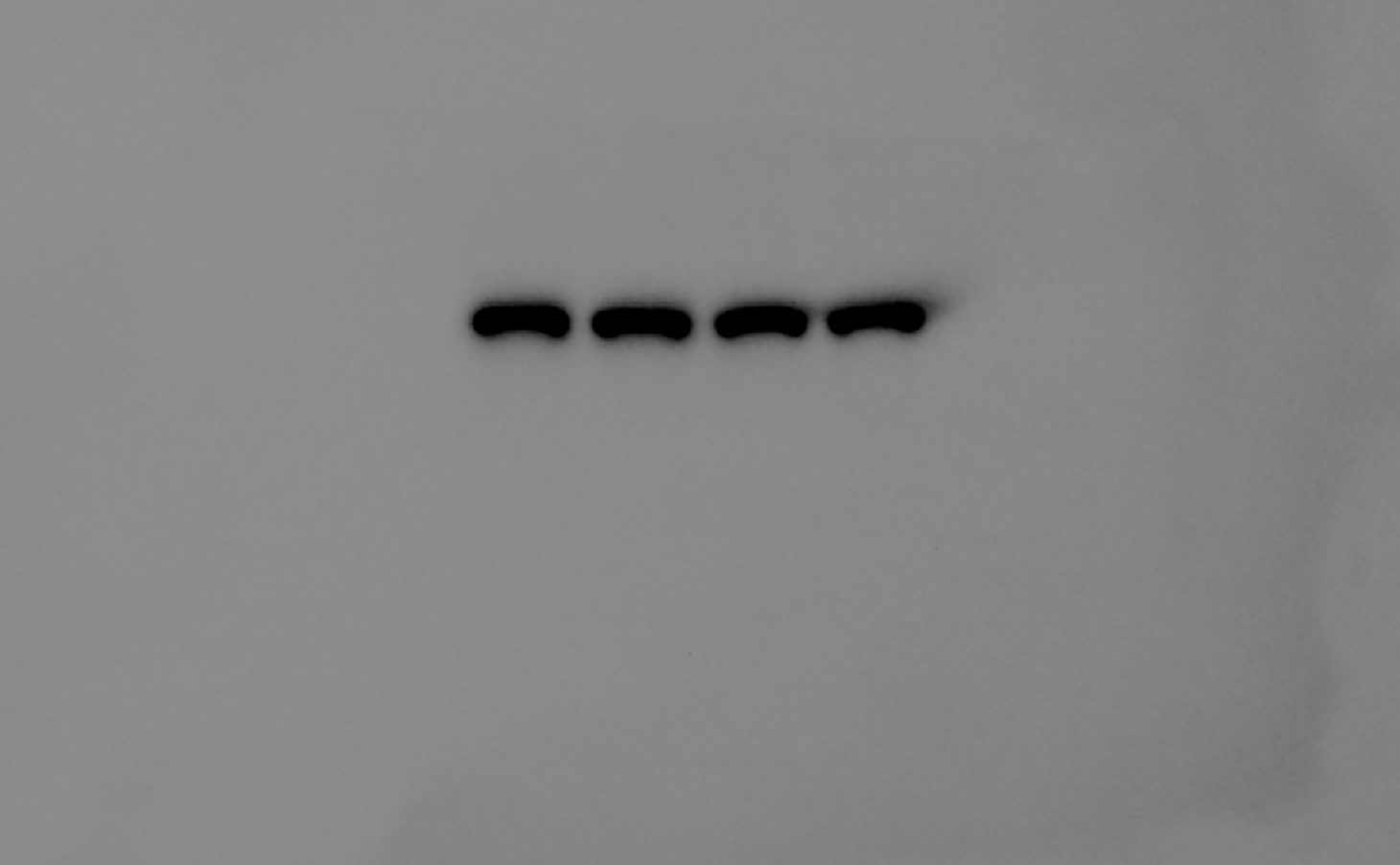


fig2C-SOCS3-1

fig2C-SOCS3-2

fig2C-SOCS3-3


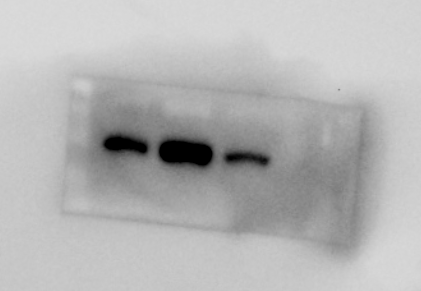

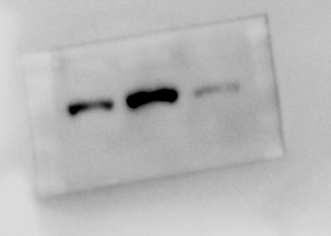

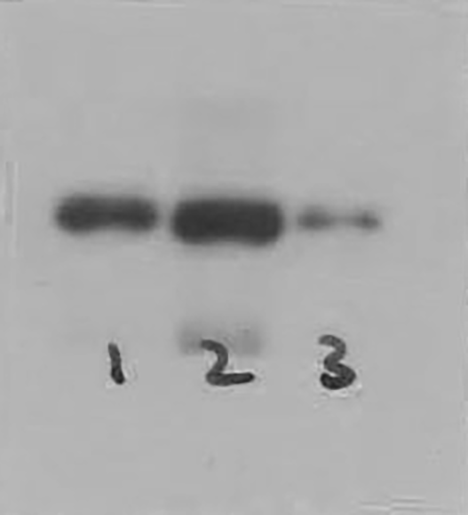


fig2C-GAPDH-3

fig2C-GAPDH-1

fig2C-GAPDH-2


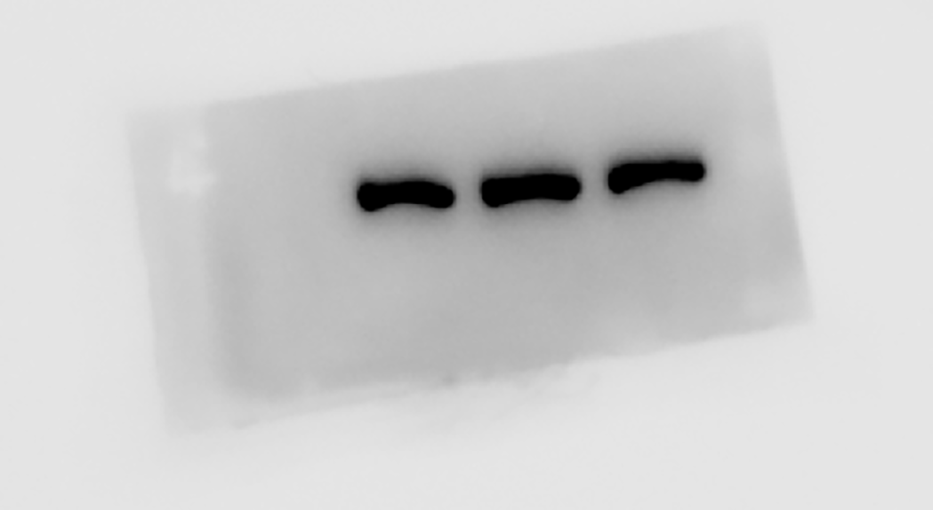

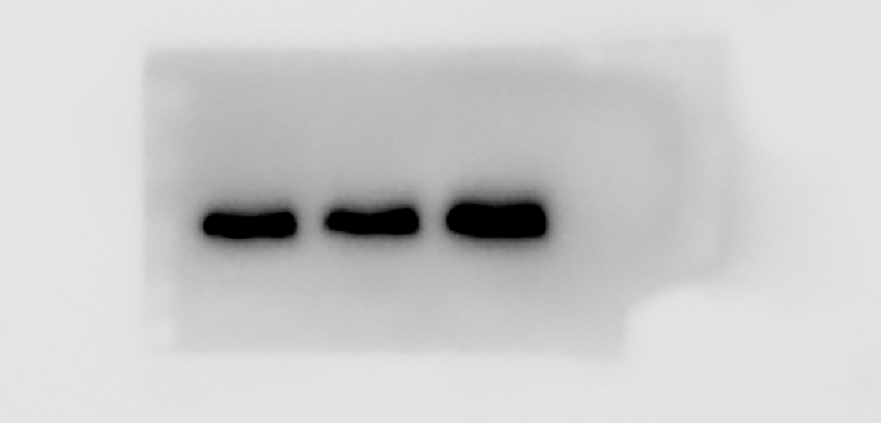

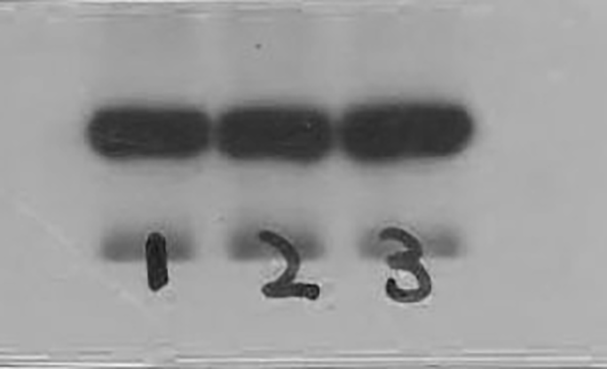

Supplement: Supplementary file 1 — Additional file 1. Uncropped full-length gels and blot results of SOCS3. [file 13287_2023_3347_MOESM1_ESM.doc]
